# Influence of the Phagemid PfNC7401 on Cereulide-Producing *Bacillus cereus* NC7401

**DOI:** 10.3390/microorganisms10050953

**Published:** 2022-04-30

**Authors:** Peiling Geng, Yunfei Gong, Xiaofu Wan, Xiaomin Hu

**Affiliations:** 1 College of Life Science, South-Central Minzu University, Wuhan 430074, China; pl.geng@siat.ac.cn (P.G.); 2020110293@mail.scuec.edu.cn (Y.G.); 2 Wuhan Institute of Virology, Chinese Academy of Sciences, Wuhan 430071, China; wanxf15@126.com

**Keywords:** prophage, *Bacillus cereus*, cereulide, transcriptome, BIOLOG, phenotype, host adaption

## Abstract

A phagemid-cured strain, NC7401-∆Pf, was constructed to survey the biological function of the plasmidal prophage PfNC7401 in cereulide-producing *Bacillus cereus* NC7401. The transcriptome analysis between the mutant and the wild strains revealed a series of differentially expressed genes mainly involved in different function classifications, including the two-component signal transduction system, bacterial structure, transporters, related antibiotic response, purine biosynthesis, non-ribosomal peptide synthetases (NRPS) and related secondary metabolites, and aromatic or other amino acid synthesis. BIOLOG and phenotypic experiment analyses confirmed that PfNC7401 may affect phage immunity and the metabolism of several amino acids, including L-Alanine, which was suggested to be related to one precursor (D-Alanine) of cereulide synthesis. However, neither the transcription levels of the cereulide production-related genes (e.g., *ilvB*, *cesA*, *cesB*, and *cesH*) nor the cereulide production nor cell cytotoxicity were affected by the presence or absence of PfNC7401, corresponding with the transcriptome data, in which only four genes unrelated to cereulide synthesis on the plasmid-carrying *ces* gene cluster were affected by the curing of PfNC7401.

## 1. Introduction

Prophages present as nucleic acids form, which are normally integrated into host replicons such as chromosomes and plasmids or exist freely such as autonomous replicating plasmids (named phagemid) [1,2,3,4]. A few prophages retain intact genetic materials and can be induced into a lytic cycle under certain conditions; a few have lost the function involved in replication and packaging for infective virions, but remain as discontinuous phage DNA in the resident host replicon, harboring complete or incomplete coding sequences and stably passing together with chromosomes or plasmids [5].

The coexistence of prophages within a bacterium might have developed as a mutually beneficial symbiosis. On one hand, the host provides a habitat for the prophage and shares its replication or other genetic devices, helping to maintain the reproduction and stability of the phage population [2]. On the other hand, the prophage endues new genes (e.g., antibiotic resistance and toxin- or immune-related genes) to the host, probably enhancing the pathogenicity and colonization of the pathogenic bacteria [4,6,7,8,9,10,11,12,13]. A prophage may also enhance the adaptation of the host to ecological niches by regulating the transcription of metabolism or fitness-related factors [14,15]. For instance, the lysogeny of a prophage on *Bacillus thuringiensis* was found to have influences on energy metabolism, growth, and the production of insecticidal crystal proteins (ICPs) [16,17].

*Bacillus cereus* is ubiquitous in nature. It is a common food spoilage and opportunistic pathogenic bacterium that can cause two types of food poisoning with symptoms of diarrhea and vomiting [18]. The latter is caused by a heat-stable peptide toxin named cereulide and may be acute (onset within 0.5~6 h after ingestion) and serious (even lethal) [19,20,21]. Cereulide is a dodecadepsipeptide consisting of three repeating units of [D-O-Leu-D-Ala-L-O-Val-L-Val] [22]. It is enzymatically synthesized via non-ribosomal peptide synthetases (NRPS) encoded by a ca. 24 kb *cesHPTABCD* gene cluster, which displays different genomic locations and is present in large plasmids with different sizes or in chromosomes [23]. The production of cereulide correlates with nutrient ingredients and the growth phase of the bacteria [24,25,26,27,28]. Cereulide is produced from the log-phase and reaches a maximal accumulation at the stationary phase during the bacterial growth. The production is, therefore, regulated by the sporulation transition factors ArbB and Spo0A as well as the global regulator of carbon and nitrogen metabolism CodY [25,29,30]. Furthermore, it can be negatively regulated by the hydrolase CesH [31].

A previous study compared the genomes of phages derived from emetic *B. cereus* strains and found they have similar structures and core genes, even if they are in a circular or a linear DNA form or with different sizes [32]. This corresponds with their host spectrum specificity and suggests that there is a coevolutionary relationship between the prophage and the host. Whether the prophage in emetic *B. cereus* is associated with the pathotypes and ecotypes of the host remains unclear.

PfNC7401 (once thought to be a circular plasmid pNC1 in NC7401) is a phagemid identified in cereulide-producing *B. cereus* isolate NC7401, which has a self-replicating capability in the lysogenic state and can produce active phages through induction [32]. In this study, a PfNC7401-cured mutant was constructed and the transcriptome and phenotype of the wild and the mutant were compared. We provide a theoretical basis for understanding the interaction and coevolution between the phagemid and the emetic *B. cereus*.

## 2. Materials and Methods

### 2.1. Bacterial Strains, Plasmids, and Primers

The bacterial strains and plasmids used in this study are listed in Table S1. NC7401 is an emetic *B. cereus* and was previously isolated [33]. *Escherichia coli* JM109 was used as the host for the construction of the recombinant plasmids. The primers used in this study are listed in Table S2.

### 2.2. Construction of Phagemid-Cured Mutant

**Figure 1:**
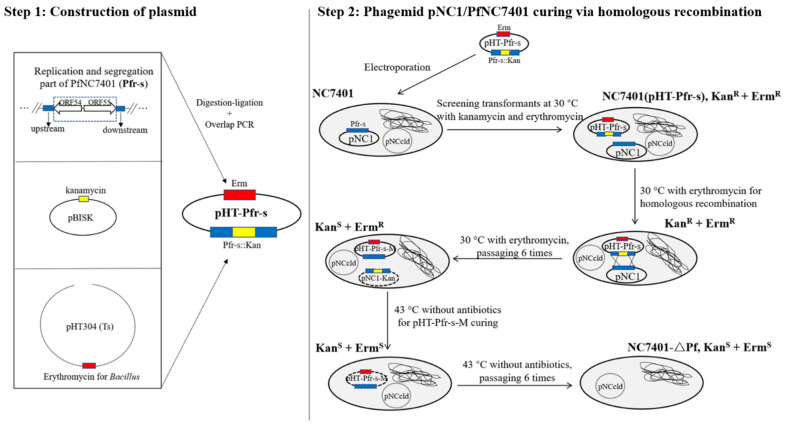
The schematic diagram of NC7401-ΔPf construction. Step 1: A recombinant plasmid pHT-Pfr-s was constructed, which consisted of a temperature-sensitive vector pHT304 (Ts) carrying erythromycin resistance, two homologous fragments of the replication (CDS55) and partition (CDS54) genes of PfNC7401 (=pNC1), and a kanamycin resistance gene marker. Step 2: pHT-Pfr-s was electroporated into NC7401-competent cells. The obtained transformants containing pHT-Pfr-s were resistant to both erythromycin and kanamycin. One colony with double resistance was picked and grown at 30 °C in LB medium with only erythromycin. At least six times of passage on growth condition with only erythromycin addition were conducted for homologous recombination. During this process, the obtained pNC1-Kan (=PfNC7401-Kan) was lost due to the replacement of a DNA fragment containing replication and partition genes by the kanamycin gene. Another six times of consecutive passage at 43 °C in LB medium without antibiotic addition were then performed for the elimination of the recombinant plasmid pHT-Pfr-s-M, which carried a temperature-sensitive suicide gene. Finally, the colonies that were sensitive to both erythromycin and kanamycin were picked as candidate phagemid-cured mutants for further PCR verification.

A homologous recombination strategy of knocking out genes related to replication (CDS55) and partition (CDS54) in the phagemid PfNC7401 (=pNC1) was carried out to construct the mutant NC7401-∆Pf (Figure 1). Primer sets PfL-F/PfL-R and PfR-F/PfR-R were used to amplify two fragments (each about 800 bp) containing the upstream and downstream sequences of PfNC7401_CDS54~55; primers Kan-F/Kan-R were used for ca. 1.3 kb of a kanamycin resistance gene marker from a lab-stored plasmid pBISK. The PCR products were purified, digested, and cloned into the temperature-sensitive vector pHT304 (Ts) together. The resulting recombinant plasmid pHT-Pfr-s was electroporated into NC7401-competent cells, followed by screening on Luria–Bertani (LB) agar with erythromycin (5 μg/mL) and kanamycin (10 μg/mL). A transformant colony was picked and then six times of passage grown at 30 °C in an LB medium with erythromycin (5 μg/mL) were conducted for the homologous recombination. During this process, the obtained pNC1-Kan was lost due to the replacement of a DNA fragment containing replication and partition genes by the kanamycin gene. Another six times of consecutive passage at 43 °C in an LB medium without an antibiotic addition were then performed for the elimination of the recombinant plasmid pHT-Pfr-s-M, which carried a temperature-sensitive replicon. Finally, the colonies that were sensitive to both erythromycin and kanamycin were picked as the candidate phagemid-cured mutants.

Two primer pairs, Portal-F/R and TLS-F/R, designed based on the sequences of DNA packaging-related genes located in PfNC7401, were used for the verification of the elimination. Furthermore, to analyze whether the use of high-temperature passaging caused the loss of the plasmid (pNCcld) harboring the cereulide biosynthesis gene cluster, cesB-Em-F1/R1 and ces-F1/R2 were used to check for the presence of *ces* cluster genes; RepX-F/R and ParA-F/R were used to detect the replication and segregation-related genes of pNCcld, respectively.

### 2.3. Large Plasmid Profile Analysis

Large plasmids were extracted as described previously [34]. The aqueous phase containing the plasmids was analyzed using 0.6% agarose gel (Seakem Gold, Lonza, Quakertown, PA, USA, Catalog: 50152) with 1 × TBE buffer in a precooled electrophoresis chamber at a voltage of 10 v/cm for 4–5 h. The gel was stained with 1 μg/mL of ethidium bromide for 15 min and destained in demineralized water at 4 °C overnight before imaging.

### 2.4. RNA Extraction and Reverse Transcription PCR (qRT-PCR)

The RNA extraction was performed with TRIzol as previously described, but with modifications [35]. In brief, cells grown in an LB medium at different growth phases were collected, resuspended with 1 mL of TRIzol, and treated with 200 μL of chloroform. The aqueous solution containing the RNA and the organic phase were separated by centrifugation (12,000× *g*, 4 °C, 15 min). Subsequently, the RNA was precipitated with an equal volume of isopropanol and rinsed with 75% alcohol. After being air-dried, the RNA was dissolved in a TE buffer (10 mM Tris, 1 mM EDTA, pH 8.0) and kept on ice or at −70 °C.

The transcriptional levels of the phagemid-related gene (CDS29 coding portal protein), the host cereulide synthesis-related genes *cesA*, *cesB*, *cesH*, and *ilvB*, and the housekeeping gene *ccpA* at different growth phases (8 h, 12 h, 24 h, and 36 h) were investigated with the primers Portal-RF-F/R, cesA-RT-F/R, cesB-RT-F/R, cesH-RT-F/R, ilvB-RT-F/R, and ccpA-RT-F/R, respectively. The relative mRNA levels were conducted by a Bio-Rad iQ2 real-time PCR detection system and a One Step TB Green™ PrimeScript™ PLUS RT-PCR Kit (Takara, Dalian, China, Catalog: RR096A) with RNAs as templates and *ccpA* as an internal standard. Three parallel and independent replicates were carried out.

### 2.5. CDNA Library Construction, RNA Sequencing, and Data Analysis

The total qualified RNA (5 μg/sample, OD_260/280_ > 1.8) with rRNA removed (Ribo-off rRNA Depletion Kit (Bacteria), Vazyme, Nanjing, China, Catalog: N407-01) was prepared from three independent biological replicates for each strain, which was extracted from the host bacteria NC7401 or NC7401-ΔPf cultured in an LB medium for 12 h. The construction of a cDNA library and the RNA sequencing were performed by Wuhan SeqHealth Tech Co., Ltd. (Wuhan, China) based on an Illumina Miseq UID-Total-RNA-seq PE150. The obtained raw reads were submitted for a quality evaluation using FastQC v.0.11.5 (http://www.bioinformatics.babraham.ac.uk/projects/fastqc/, accessed on 1 March 2022) and then filtered by Trimmomatic (version 0.36) in which the adaptor sequences were trimmed and the reads with a low quality were discarded. After UID deduplication, about 1 G of total clean reads were yielded for each biological sample. The data were mapped to the reference genome of NC7401 (GCF_000283675.1_ASM28367v1) to obtain the global transcript information, including the distribution of reads and the gene expression level. The gene expression was measured with the RPKM (reads per kilobase per million reads) with the following equation: RPKM = (total exon reads)/([mapped reads (millions) × exon length (kb)]). A Spearman correlation coefficient was used to evaluate the biological repetition. Differential expression genes were marked with ∣logFC∣ > 1 and a *p*-value < 0.05, where the logFC was log2(FC) and FC indicated the fold change of the RPKM of the same gene in different samples. The top 20 differentially expressed genes according to the *p*-value were chosen to conduct the gene ontology (GO) and Kyoto Encyclopedia of Genes and Genomes (KEGG) pathway enrichment analyses and to construct the enriched bubble charts.

### 2.6. BIOLOG Phenotype Microarray Analysis

To evaluate the phenotype differences, BIOLOG phenotype microarray experiments with PM3B nitrogen sources and PM17A antibiotic and chemical substances were performed on an OminoLog Incubator-Reader following the manufacturer’s instructions. All PM plates were incubated at 28 °C and automatically monitored every 15 min for 45 h. The BIOLOG data were analyzed and visualized using OminoLog PM software (OL_PM_FM/Kin 1.30) based on the differences of the growth rates of the tested bacteria, which were indicated by the reduction of tetrazolium violet to purple formazan with the active metabolism of the cells and recorded by a charge-coupled-device camera. All experiments were carried out in duplicate. The metabolism differences were calculated by the mean difference between the data at 45 h and at 0 h. A BIOLOG analysis was conducted by Stamp [36].

### 2.7. Bacterial Growth, Motility, and Biofilm Analysis

The overnight culture of the tested bacteria was transferred into 50 mL of a fresh LB medium or a CADM medium with or without L-Phenylalanine or/and L-Alanine at a ratio of 1:1000, respectively. The bacterial absorbances at 595 nm were monitored at intervals during a growth period of about 60 h at 30 °C.

A total of 3 μL of overnight bacterial culture in an LB medium was spotted onto an LB semi-solid medium (0.5% agar) and cultured at 30 °C for 48 h. The bacterial mobility was measured by the diameter of the spot.

The biofilm formation was determined by the crystal violet method. The overnight bacterial culture in an LB medium was diluted with an LB medium at a ratio of 1:100; 200 μL was then cultured in a 96-well plate at 30 °C for 48 h. The bounded cells were gently washed twice with distilled H_2_O and stained with 250 μL 0.1% (*w*/*v*) aqueous solution of crystal violet for each well for 30 min at room temperature and gently washed three times with distilled H_2_O. After being dried for 1 h, the crystal violet dye was solubilized with 225 μL absolute ethanol and the absorbance was measured at 600 nm.

All the experiments above were carried out in triplicate and the related statistical analysis was conducted by a *t*-test.

### 2.8. Immunity and Resistance Analysis

The bacterial sensitivity to the phage infection was determined with the drop-spot method. A total of 10 μL of 10-fold dilution phage solutions (titer > 10^5^ PFU/mL) was spotted onto the corresponding bacterial soft agar (0.5%) to observe the lysis zones after incubation at 30 °C for 12 h.

The overnight bacterial cultures were inoculated at a ratio of 1:1000 into 200 μL of a fresh LB medium mixed with antibiotic solutions at different concentrations (ampicillin 10, 25, 50, and 100 μg/mL; kanamycin 5, 12.5, 25, and 50 μg/mL; spectinomycin 10, 25, 50, and 100 μg/mL; streptomycin sulfate 10, 25, 50, and 100 μg/mL; tetracycline 1, 2.5, 5, and 10 μg/mL; chloramphenicol 2.5, 7.5, 12.5, and 25 μg/mL; mitomycin C 0.1, 0.25, 0.5, and 1 μg/mL; and erythromycin 2.5, 7.5, 12.5, and 25 μg/mL) in 96-well plates and the LB medium mixed with equal amounts of bacteria without antibiotics was used as the control. After incubation at 30 °C for 24 h, the inhibition rates were calculated via the following equation: inhibition percentage (%) = (OD_595_ of bacteria without antibiotics − OD_595_ of bacteria with antibiotics)/OD_595_ of bacteria without antibiotics × 100%.

All the experiments above were carried out in triplicate.

### 2.9. Siderophores Synthesis Ability Analysis

The siderophores synthesis ability was determined by 10 × CAS solution (chrome azurol sulphonate 0.06 g/L, FeCl_3_.6H_2_O 0.0027 g/L, and hexadecyl-ltrimethyl-ammonium bromide 0.073 g/L) according to the handbook (Coolaber, Beijing, China, Catalog: PM0821-2). Briefly, after incubation in an LB medium at 30 °C overnight, the supernatant of the bacterial culture (OD_595_ = 0.8~1.0) was collected and mixed with an equal volume of a CAS detection solution and kept in the dark for 30 min; the absorbance at 630 nm (As) was then measured. An LB medium mixed with a CAS solution without bacteria was used as the control and the absorbance at 630 nm was measured as Ar. The relative siderophore content was calculated using the following formula: relative siderophore content (%) = [(Ar − As)/Ar] × 100%. The experiments were carried out in triplicate and analyzed by a *t*-test.

### 2.10. Cereulide Production and Cytotoxicity Assay

The toxin cereulide was extracted using methanol and semi-quantified using high-performance liquid chromatography (HPLC) as previously described [31,37,38]. Valinomycin as a structural analog of cereulide was used as a reference to determine the concentration of the extracted cereulide. The HPLC was conducted with a WelchLP-C18 chromatographic column (4.6 × 250 mm, 5 μm particle size) equipped with an online filter. The mobile phases were 0.1% glacial acetic acid and 100% methanol (5%; 95%, *v*/*v*). After washing and an equilibrium at a flow rate of 1 mL/min for 30 min, the absorbance at 210 nm was monitored. The results were analyzed via Chromeleon 7 software.

A cytotoxicity test of the cereulide was carried out using human liver cancer cells HepG2 [39]. The cells were recovered and passaged in Dulbecco’s Modified Eagle Medium (DMEM) with 10% fetal bovine serum, penicillin (100 U/mL), and streptomycin (0.1 mg/mL) at 37 °C with 5% CO_2_ and MTT (3-(4,5-Dimethylthiazol-2-yl)-2,5-diphenyltetrazolium bromide) A cytotoxicity assay experiment was conducted according to the manual (Beyotime, Shanghai, China, Catalog: C0009S). Each well, which contained ca. 5000 cells, was supplemented with 1 μL of a cereulide crude extraction or the same amount of methanol as a negative control in a 96-well plate. The cytotoxicity of the cereulide was measured by A570 with a microplate reader with the following equation: cell viability = (1 − (OD of methanol added − OD of cereulide added)/(OD of methanol added)) × 100%.

All the experiments above were carried out in triplicate and the statistical analysis was conducted by a *t*-test.

## 3. Results and Discussion

### 3.1. Construction of the Phagemid-Cured Mutant NC7401-∆Pf

As PfNC7401 (=pNC1) is a “plasmidal” prophage [32], the phagemid-cured mutant NC7401-∆Pf was constructed by disrupting its replication capability via the homologous recombination knock-out of the replication/segregation-related region (Figure 1). A large plasmid profile indicated that, compared with the wild NC7401, NC7401-∆Pf lost PfNC7401 whereas another large plasmid, pNCcld, remained, harboring a cereulide biosynthesis gene cluster (Figure S1a). This was further verified by the PCR results in which NC7401-∆Pf displayed a negative result for the detection of phage DNA packaging-related genes located in PfNC7401 and a positive result for the detection of a *ces* cluster and replication/segregation-related genes located in pNCcld (Figure S1b).

### 3.2. Transcriptome Overview of NC7401 and NC7401-∆Pf

**Figure 2:**
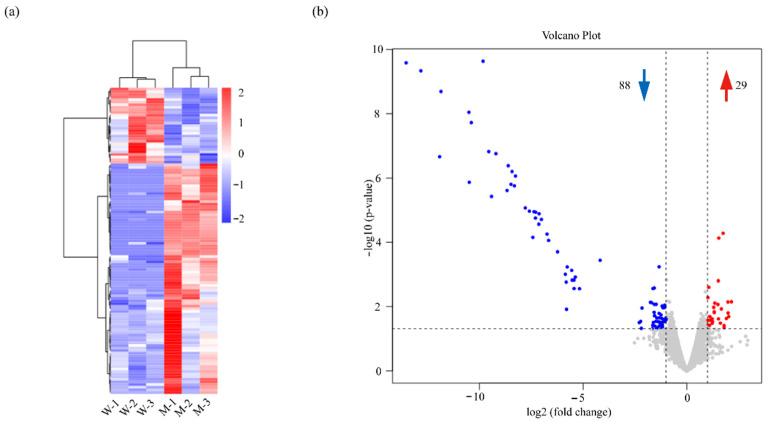
Overview of the differential expression genes in NC7401 and NC7401-ΔPf. (**a**) Heatmap of the differential expression genes. Hierarchical clustering diagram of all the differential expression genes was created based on RPKM. Blue and red indicate genes expressed at low and high levels, respectively. (**b**) Volcano plot of the differential expression genes. Grey dots indicate genes with no differential expression whereas blue and red dots represent the downregulated and upregulated genes, respectively.

The RNA-seq identified a series of genes with a differential expression, of which 88 genes were downregulated and 29 were upregulated in NC7401-∆Pf compared with NC7401 (Figure 2). These genes mainly belonged to “moderate” (RPKM values that ranged from 10~100) and “low” (RPKM values that ranged from 1~10) expression levels (Table S3).

**Figure 3:**
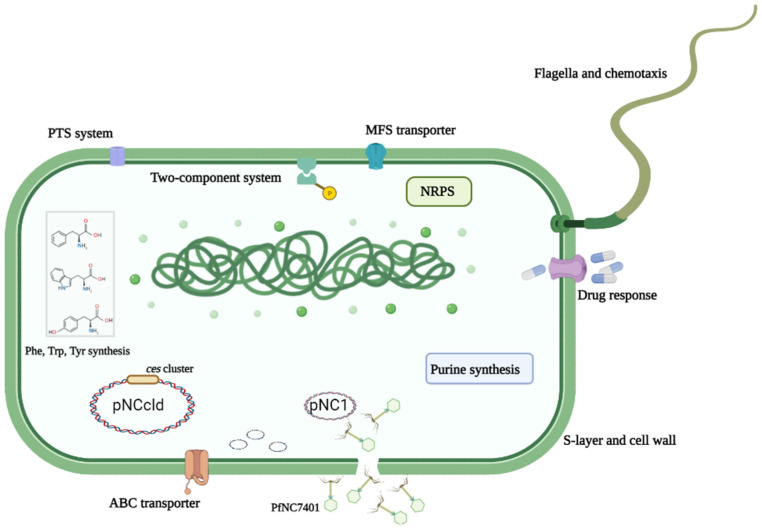
Putative influenced pathways of PfNC7401/pNC1 on the host. Host cell metabolic pathways and different structures related to differentially expressed genes are indicated in the diagram with different colors or boxes.

In total, 28 upregulated and 46 downregulated genes were in the chromosome (Table S4). A KEGG pathway analysis indicated that the downregulated genes were involved in the biosynthesis of a siderophore group of non-ribosomal peptides and ABC transporters whereas the upregulated genes were for phenylalanine, tyrosine, and tryptophan biosynthesis as well as the biosynthesis of amino acids (Figure S2). This was in line with the GO-enriched gene function analysis (Figure S3). These differentially expressed genes could be mainly involved in different function classifications, including the two-component signal transduction system, bacterial structure, transporters, related antibiotic response, purine biosynthesis, related NRPS and secondary metabolites, and aromatic or other amino acid synthesis (Table S5, Figure 3). Generally, the knock-out of even only one or several important chromosomal gene(s) may cause transcriptional changes in hundreds of genes. The curing of the complete PfNC7401, however, caused much fewer changes. It indicated that PfNC7401 was an independent episome.

Only one gene (BCN_RS21365, encoding germination protease) in pNCcld was upregulated and three (two uncharacterized protein encoding genes, BCN_RS27720 and BCN_RS28415, and one ArsR transcriptional regulator, BCN_RS28400) were downregulated in NC7401-∆Pf (Table S4). This indicated that the effects of PfNC7401 on the plasmid-carrying *ces* gene cluster were limited.

**Figure 4:**
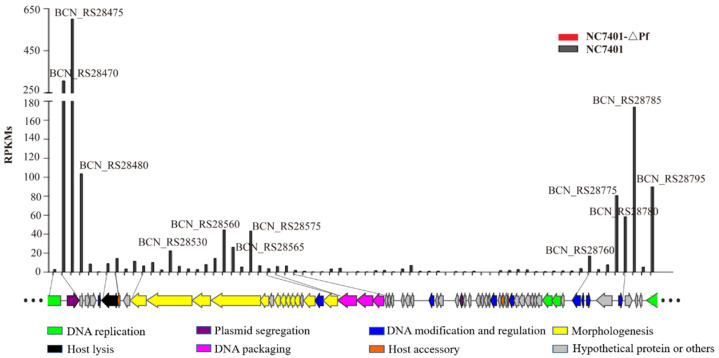
RNA-seq data comparison of PfNC7401 in NC7401 and NC7401-∆Pf. Genes of PfNC7401 are represented by arrowhead boxes and colored according to their putative functions. RPKMs of all genes are plotted above the phage diagram with different colored columns. RPKM was used to measure the amount of gene expression with the following equation: RPKM = (total exon reads)/([mapped reads (millions) × exon length (kb)]). No gene encoded by PfNC7401 was transcribed in NC7401-ΔPf whereas for the wild strain itself, 39 genes of PfNC7401 displayed different transcriptional levels.

We did not find any gene encoded by PfNC7401 that was transcribed into the cured strain. For the wild strain itself, 39 genes of PfNC7401 displayed different transcriptional levels, of which 4 and 12 showed high and moderate expression levels whereas 23 showed low expression levels (Table S6). These genes are involved in plasmid replication (BCN_RS28465, BCN_RS28745–BCN_RS28750) and partition (BCN_RS28470), lysogenic-related (BCN_RS28760–BCN_RS28780), DNA packaging (BCN_RS28590–BCN_RS28600), phage lysis (BCN_RS28495–BCN_RS28500), phage morphogenesis (BCN_RS28515–BCN_RS28585), and a few hypothetical proteins (Figure 4). The other 29 genes of PfNC7401 exhibited a silent status in NC7401 (Table S6). This was probably due to the lysogenic state of PfNC7401 in NC7401 or the limitation, as we only analyzed the transcriptome data for one time point (12 h).

### 3.3. Effects of the Curing of PfNC7401 on Growth and Metabolism

**Figure 5:**
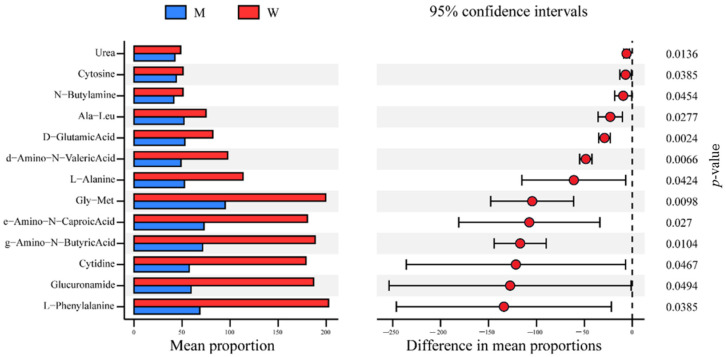
BIOLOG analysis of NC7401 and NC7401-∆Pf showing the differences of metabolic capability of different nitrogen sources via PM3B using Stamp. M and W indicate phagemid-cured strain NC7401-ΔPf and the wild strain *B. cereus* NC7401, respectively. The “mean proportions” (**left**) were calculated by the mean values of bacterial metabolic differences (data at 45 h–data at 0 h), which were indicated by the reduction of tetrazolium violet to purple formazan with active metabolism of cells and recorded by a charge-coupled-device camera. The “difference in mean proportions” (**right**) indicated the difference degrees of two bacteria using different substrates within 95% confidence intervals. Dots indicate the difference of mean proportions (mutant minus wild strain).

**Figure 6:**
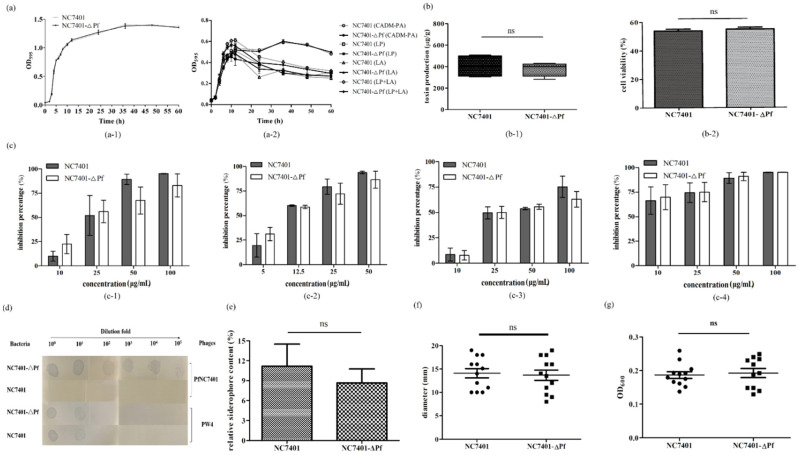
Influence of phagemid elimination on bacterial physiological and biochemical features. (**a**) Growth curves of NC7401 and NC7401-ΔPf in LB medium (**a-1**) and in CADM medium with or without 0.1 g/L of L-Phenylalanine or/and L-Alaninemedium (**a-2**). (**b**) Cereulide production of NC7401 and NC7401-ΔPf at sporulation stage (**b-1**) and cytotoxicity assay of HepG2 cells (**b-2**). (**c**) Sensibility assay of NC7401 and NC7401-ΔPf to different antibiotics at different concentrations. (**c**-**1**–**c**-**4**) represent inhibition rates against NC7401 and NC7401-ΔPf of ampicillin, kanamycin, spectinomycin, and streptomycin sulfate, respectively. (**d**) Lysis zones formed by PfNC7401 and PW4 on the bacterial lawns of NC7401 and NC7401-ΔPf, respectively. (**e**) Relative siderophore content comparison of NC7401 and NC7401-ΔPf using CAS method. (**f**) Mobility and (**g**) biofilm production ability of NC7401 and NC7401-ΔPf.

A PM3B BIOLOG analysis showed that the utilization of several nitrogen sources (e.g., L-Phenylalanine, Glucuronamide, Cytidine, g-Amino-N-Butyric Acid, e-Amino-N-Caproic Acid, Gly-Met, and L-Alanine) sharply declined in NC7401-ΔPf compared with the wild strain (Figure 5 and Table S7). This corresponded with the transcriptome data to a degree, which reported a few affected genes involved in amino acid biosynthesis. However, the elimination of PfNC7401 had no obvious effect on the bacterial growth in the LB medium nor in a full synthetic CADM medium with or without L-Phenylalanine or/and L-Alanine (Figure 6a). Our data suggested that although the curing of PfNC7401 led to the decreased utilization of several nitrogen sources, in turn, the lack of these nitrogen sources did not affect the growth of the mutant. This phenomenon indicated that there were bypassed metabolic pathways that compensated for these nutritional deficiencies.

Remarkably, we observed that the utilization of L-Ala sharply declined in NC7401-ΔPf compared with the wild strain. The precursors of D-O-Leu, D-Ala, and L-O-Val of the cereulide were thought to be related to the branched chain amino acids, L-Ala, L-Val, and L-Leu [40]. However, Agata et al. found that only valine, leucine, and threonine were essential for growth and cereulide production by *B. cereus* [41]. Nevertheless, Kuse et al. observed that L-Ala could also be incorporated into the carboxylic carbon atoms of D-Ala of cereulide at a much lower percentage than L-Leu and L-Val into those of D-O-Leu and L-O-Val [40]. Considering that only pyruvic acid might be diluted due to a high amount of stock because L-Ala is not essential to *B. cereus,* Kuse et al. speculated that all three L-amino acids might be converted into keto acids before being reduced to D-O-Leu and L-O-Val or being transaminated to D-Ala [40]. This may explain why the elimination of PfNC7401 had no obvious effect on the bacterial growth in the CADM medium with or without L-Ala in our study.

Furthermore, the HPLC and MTT results indicated that neither the cereulide production nor cell cytotoxicity were affected by the presence or absence of PfNC7401 (Figure 6b). The transcription levels of the cereulide production-related genes (e.g., *ilvB*, *cesA*, *cesB*, and *cesH*) between the wild strain and the prophage-cured strain did not display obvious differences (Figure S4). This corresponded with the transcriptome data in which only four genes unrelated to cereulide synthesis on the plasmid-carrying *ces* gene cluster were affected by the curing of PfNC7401.

### 3.4. Effects of the Curing of PfNC7401 on Immunity and Resistance

As the transcriptome data revealed that several genes related to a multidrug-resistant-related response (e.g., BCN_RS07505, BCN_RS09675, BCN_RS20820, BCN_RS24760, and BCN_RS15620) were downregulated in the mutant compared with the wild strain (Table S5), their immunity and resistance were analyzed. No significant differences (*p* < 0.05) against the tested antibiotics and chemical substances (e.g., Sodiumsalicylate, Cefsulodin and D-Serine) between NC7401 and NC7401-ΔPf were observed (Figure S5 and Table S8) by the PM17A BIOLOG analysis. Other antibiotics were tested by inhibition experiments. The results showed that NC7401-∆Pf seemed to be slightly more sensitive to β-lactam-type antibiotic ampicillin and aminoglycoside-type antibiotic kanamycin when at a low dose (10 and 5 μg/mL, respectively) than NC7401 whereas an opposite effect was displayed when at higher concentrations (50~100 and 25~50 μg/mL, respectively). For another two aminoglycoside-type antibiotics, PfNC7401 provided the bacteria with greater resistance against spectinomycin only at 100 μg/mL and had no influence at lower concentrations whereas resistance against streptomycin sulfate was dose-dependent in both the wild strain and the prophage-cured strain, but with no difference at the same concentration (Figure 6c). Generally, the differences in these tested antibiotics were slight and were not statistically significant. No obvious sensitivity differences against the other tested antibiotics (e.g., tetracycline, chloramphenicol, erythromycin, and mitomycin C) were observed (Figure S6).

Compared with the wild strain displaying immunity to PfNC7401, NC7401-∆Pf became sensitive after curing this endogenous prophage whereas another *B. cereus* phage, PW4 [42], showed a similar infective level against both NC7401 and NC7401-∆Pf (Figure 6d). Neither the similar replication gene nor other large genome contents were found to be shared by PW4 and PfNC7401 [42]. This may explain why PW4 could escape the phage immunity from NC7401.

### 3.5. Effects of the Curing of PfNC7401 on Other Phenotypes

The transcriptome data revealed that a few genes related to siderophore group NRPS (e.g., 2,3-dihydro-2,3-dihydroxybenzoate dehydrogenase BCN_RS11725 and isochorismatase BCN_RS11740) were declined (Table S5). The quantitative activity units of the siderophore were slightly reduced in the mutant compared with the wild type (8.67% vs. 11.17%) after growth for 12 h in an LB medium, but the difference was not significant (Figure 6e). The siderophore synthesis ability of the wild strain was originally very low (As/Ar ≈ 0.899).

Although the transcriptome data also revealed that several genes related to two-component signal transduction systems (e.g., CheY-homologous receiver protein BCN_RS02260 and chemotaxis family protein BCN_RS07510) and arginine metabolism proteins related to sporulation (e.g., BCN_RS19510 (stage V sporulation protein AD) and BCN_RS19520 (stage V sporulation protein AB)) were affected (Table S5), neither the mobility (Figure 6f) nor biofilm production (Figure 6g) nor sporulation ratio (data not shown) of NC7401 and NC7401-∆Pf showed significant differences. The inconsistence between the transcriptome data and these phenotype experiments was probably due to (1) the fact that we only analyzed the transcriptome data for one time point (12 h) whereas we recorded the biofilm/mobility and sporulation data at different time points (48 h for the former two and 120 h for the latter); and (2) the methods used in the phenotypic experiments were limited. Reference [43] is cited in the Supplementary Materials.

## 4. Conclusions

The elimination of PfNC7401 caused a transcriptional difference between the mutant and the wild strains within the genes mainly involved in different function classifications, including the two-component signal transduction system, bacterial structure, transporters, related antibiotic response, purine biosynthesis, related NRPS and secondary metabolites, and aromatic or other amino acid synthesis (Figure 3). The phenotypic experiment analyses revealed that PfNC7401 could affect phage immunity and the metabolism of several amino acids, including L-Alanine, which is probably related to one precursor (D-Alanine) of cereulide synthesis to a degree. PfNC7401 was also found to display a slight influence on antibiotic resistance and siderophore synthesis, but the differences were not statistically significant. However, several other phenotypes (growth, mobility, biofilm formation, and sporulation rate) were not affected, inconsistent with the transcriptome analysis. The discrepancies of the transcriptomic data with these phenotypic analyses could result from the different time points for data mining (e.g., 12 h for transcriptomic vs. 48 h for biofilm and mobility and 120 h for sporulation). Furthermore, neither the transcription levels of the cereulide production-related genes (e.g., *ilvB*, *cesA*, *cesB*, and *cesH*) nor the cereulide production nor cell cytotoxicity were affected by the elimination of PfNC7401, corresponding with the transcriptome data in which only four genes unrelated to cereulide synthesis on the plasmid-carrying *ces* gene cluster were affected by the curing of PfNC7401.

## Data Availability

Not applicable.

## Supplementary Materials

The following supporting information can be downloaded at: https://www.mdpi.com/article/10.3390/microorganisms10050953/s1, Figure S1: Verification of phagemid elimination. (a) Large plasmid profile. M: Trans 15k DNA marker; Lane 1–3: plasmid profiles of AND508, NC7401, and NC7401-∆Pf. The black arrows indicate pNCcld (upper) and the phagemid pfNC7401 (lower). (b) Verification of phagemid elimination by PCR. Every six lanes were set as a group using a template prepared from the same strain in which lanes 1–6 represent PCR results for NC740 and lanes 7–12 for NC7401-∆Pf. The primer pairs used for each well in each group were Portal-F/R, TLS-F/R, cesB-Em-F1/R1, ces-F1/R2, RepX-F/R, and ParA-F/R in that order and used for detecting the occurrence of specific genes related to the phagemid or cereulide encoding plasmid. M: Trans2K DNA marker. Figure S2: KEGG pathway analysis of the differential expression genes of NC7401 and NC7401-ΔPf. The bubble charts were constructed based on the downregulated (a) and upregulated genes (b) identified by RNA-seq analysis. The significances of enrichment were measured by -log10 (*p*-value); the greater the value, the greater the significance. The size of the dot indicates the number of differential genes contained in the corresponding KEGG pathway; the bigger the size, the greater the number of involved genes. Figure S3: GO-enriched gene function analysis of downregulated (a) and upregulated genes (b) in NC7401 and NC7401-ΔPf. The value of −log10 (*p*-value) indicates the significance of enrichment; the greater the value, the greater the significance. The size of the dot represents the number of differential genes involved in the corresponding GO pathway; the bigger the size, the greater the number of involved genes. Figure S4: Effect of phagemid elimination on transcription levels of cereulide production-related genes of NC7401 and NC7401-ΔPf, respectively. The mRNA abundance differences were measured at 8 h (a), 12 h (b), 24 h (c), and 36 h (d), respectively, with *ccpA* as the reference. Figure S5: BIOLOG analysis of NC7401 and NC7401-ΔPf showing the differences of metabolic capability of various antibiotics and chemical substances via PM17A by Stamp analysis. M and W indicate prophage-cured strain NC7401-ΔPf and wild *B. cereus* strain NC7401, respectively. Figure S6: Sensitivity assay of NC7401 and NC7401-ΔPf of different antibiotics at different concentrations. (a)–(d) represent inhibition rate against NC7401 and NC7401-ΔPf of tetracycline, chloramphenicol, erythromycin, and mitomycin C, respectively. Table S1: Strains and plasmids used in this study. Table S2: Primers used in this study. Table S3: Summary of RNA levels in the whole transcriptome in wild strain NC7401. Table S4: The information of differentially expressed genes of bacterial chromosomes and pNCcld. Table S5: Pathways of differentially expressed genes involved in host adaption. Table S6: RNA-seq data of pNC1/PfNC7401 in NC7401 and NC7401-ΔPf. Table S7: Differential metabolic analysis of nitrogen sources via PM3B between NC7401 and NC7401-ΔPf. Table S8: Differential metabolic analysis of various antibiotics and chemical substances via PM17A between NC7401 and NC7401-ΔPf.
