# Supplementary material for: Influence of the Phagemid PfNC7401 on Cereulide-Producing Bacillus cereus NC7401
Source: Microorganisms. 2022 Apr 30;10(5):953. doi: 10.3390/microorganisms10050953 (PMC9143728; doi:10.3390/microorganisms10050953)
Supplement: Supplementary file 1 [file microorganisms-10-00953-s001.zip › Table S1-GPL-edied-no mark.pdf]

Table S1. Bacterial strains and plasmids used in this study.

Names	Characteristics	Sources or references
Strains		
NC7401	*B. cereus*, emetic	[33]
NC7401-△Pf	The mutant of NC7401 cured of prophage PfNC7401	This study
JM109	recA1 endA1 supE44 hsdR17 gyrA96 thi relA1 λ - △(lac-proAB)/F'[lacIq lacZ△M15 proAB+ traD36	Lab stock
Plasmids		
pHT304 (Ts)	Shuttle vector of *E. coli* and *Bacillus* with a temperature-sensitive replicon; Amp ^{R} in *E. coli* and Erm ^{R} in *Bacillus*	[43]
pBISK	Plasmid used as PCR template for kanamycin resistance gene amplification	Lab stock
pHT-Pfr-s	Recombinant plasmid for the elimination of prophage in NC7401; Kan^{R} and Erm ^{R}	This study
pMD18-T simple	TA cloning vector; Amp ^{R}	TAKARA
portal-T	pMD18-T simple with gene *portal*	This study
cesA-T	pMD18-T simple with gene *cesA*	Lab stock
cesB -T	pMD18-T simple with gene *cesB*	This study
cesH -T	pMD18-T simple with gene *cesH*	Lab stock
ilvB -T	pMD18-T simple with gene *ilvB*	Lab stock
ccpA-pHT304 (Ts)	pHT304 (Ts) with *ccpA*	Lab stock
