# Supplementary material for: Influence of the Phagemid PfNC7401 on Cereulide-Producing Bacillus cereus NC7401
Source: Microorganisms. 2022 Apr 30;10(5):953. doi: 10.3390/microorganisms10050953 (PMC9143728; doi:10.3390/microorganisms10050953)
Supplement: Supplementary file 1 [file microorganisms-10-00953-s001.zip › Table S2-GPL-edited-no mark.pdf]

Table S2. Primers used in this study.

Primers	Nucleotide sequences (5’→3’)
PfL-F	GCGTCGACTTAGGGTGTCTTTTCTTTA
PfL-R	CCCGGATCCGATGGTAGAATAGGCTTTA
PfR-F	CGGGGTACCAAATACTCATGGCATACAG
PfR-R	CCGGAATTCAAGAAAATCTTCCACAAGG
Kan-F	CCCGGATCCGAACCATTTGAGGTGATAGGT
Kan-R	CGGGGTACCGGTACTAAAACAATTCATCCAG
Portal-F	AATGGCTGCTCTTATCTTT
Portal-R	CTCCTGGACTATTATTCTGTTT
TLS-F	CCTTTAATAATCAGGCACA
TLS-R	ATAATACTGAAACGCATCG
cesB-Em-F1	GCAGCCTTCCAATTACTCCTTCTGCCACAGT
cesB-Em-R1	ATTGTACTTGCTCGTAGAAATTTCTCTTGTC
ces-F1	GGTGACACATTATCATATAAGGTG
ces-R2	GTAAGCGAACCTGTCTGTAACAACA
RepX-F	AAACGGTGTAAATGATGAAG
RepX-R	GACTAGGTATGGGTGGATGT
ParA-F	GTCTTAGCCACGATAGTAAA
ParA-R	ATGTGATGGACCTTTGTATT
portal-RT-F	TCGGTAGAATTGCGTGTTGA
portal-RT-R	TTTCGTGCGACTGCTTAGTT
cesA-RT-F	GATTACGTTCGATTATTTGAAG
cesA-RT-R	CGTAGTGGCAATTTCGCAT
cesB-RT-F	AAGCCATACGGTGACAATAC
cesB-RT-R	CAACTTACAAAGCGACAATT
cesH-RT-F	TGCTTAGTTCTTGACCTA
cesH-RT-R	CACAACAGACTTACCTTC
ilvB-RT-F	AAGCCTGATGAATTAGTTATTG
ilvB-RT-R	CTGGTTGACACGATAGTAA
ccpA-RT-F	ACTGATGGCGTTTCATTTTG
ccpA-RT-R	AAGTGGACGGCATTGTTTTC
