# Supplementary material for: Influence of the Phagemid PfNC7401 on Cereulide-Producing Bacillus cereus NC7401
Source: Microorganisms. 2022 Apr 30;10(5):953. doi: 10.3390/microorganisms10050953 (PMC9143728; doi:10.3390/microorganisms10050953)
Supplement: Supplementary file 1 [file microorganisms-10-00953-s001.zip › Table S3-GPL-edited-no mark.pdf]

Table S3. Summary of RNA levels in the whole transcriptome in the wild strain NC7401.

Expression	RKPM	Percent (%)	pNC1 (up/down)	pNCcld (up/down)	Chromosome (up/down)
extremely high	≥10000	10/5948 (0.17%)	0	0	0
very high	1000-10000	217/5948 (3.65%)	0	0	1/0
high	100-1000	961/5948 (16.16%)	0/4	0	1/2
moderate	10-100	2147/5948 (36.10%)	0/12	0/2	2/27
low	1-10	1972/5948 (33.15%)	0/23	0/1	18/17
very low or no	≤1	641/5948 (10.78%)	0	1/0	6/0
