# Supplementary material for: Influence of the Phagemid PfNC7401 on Cereulide-Producing Bacillus cereus NC7401
Source: Microorganisms. 2022 Apr 30;10(5):953. doi: 10.3390/microorganisms10050953 (PMC9143728; doi:10.3390/microorganisms10050953)
Supplement: Supplementary file 1 [file microorganisms-10-00953-s001.zip › Table S7-GPL-edited-no mark.pdf]

Table S7. Differential metabolic analysis of nitrogen sources *via* PM3B between NC7401 and NC7401-ΔPf.

OTU	NC7401	NC7401-ΔPf	Difference	P-value
L-Phenylalanine	202.5	68.5	-134	0.038536
Glucuronamide	187	59.5	-127.5	0.049444
Cytidine	179	57.5	-121.5	0.046736
g-Amino-N-Butyric Acid	188.5	71.5	-117	0.010398
e-Amino-N-Caproic Acid	180.5	73	-107.5	0.026988
Gly-Met	199.5	95	-104.5	0.009809
L-Alanine	113.5	52.5	-61	0.042397
d-Amino-N-Valeric Acid	97.5	49	-48.5	0.006563
D-Glutamic Acid	82	53	-29	0.00237
Ala-Leu	75	52	-23	0.027662
N-Butylamine	51	41.5	-9.5	0.045425
Cytosine	51	44	-7	0.038476
Urea	48.5	42.5	-6	0.013606
