# Supplementary material for: Influence of the Phagemid PfNC7401 on Cereulide-Producing Bacillus cereus NC7401
Source: Microorganisms. 2022 Apr 30;10(5):953. doi: 10.3390/microorganisms10050953 (PMC9143728; doi:10.3390/microorganisms10050953)
Supplement: Supplementary file 1 [file microorganisms-10-00953-s001.zip › Table S8-GPL-edited-no mark.pdf]

Table S8. Differential metabolic analysis of various antibiotics and chemical substances *via* PM17A between NC7401 and NC7401-ΔPf.

OTU	NC7401	NC7401-ΔPf	Difference	P-value
Sodiumsalicylate	197.5	152	-45.5	0.051136
Cefsulodin	204.5	216.5	12	0.075329
Sodiumsalicylate.1	151.5	148.5	-3	0.051317
D-Serine	203.5	198.5	-5	0.142507
