# Supplementary figures and images for: Influence of the Phagemid PfNC7401 on Cereulide-Producing Bacillus cereus NC7401
Source: Microorganisms. 2022 Apr 30;10(5):953. doi: 10.3390/microorganisms10050953 (PMC9143728; doi:10.3390/microorganisms10050953)

## Slide 1
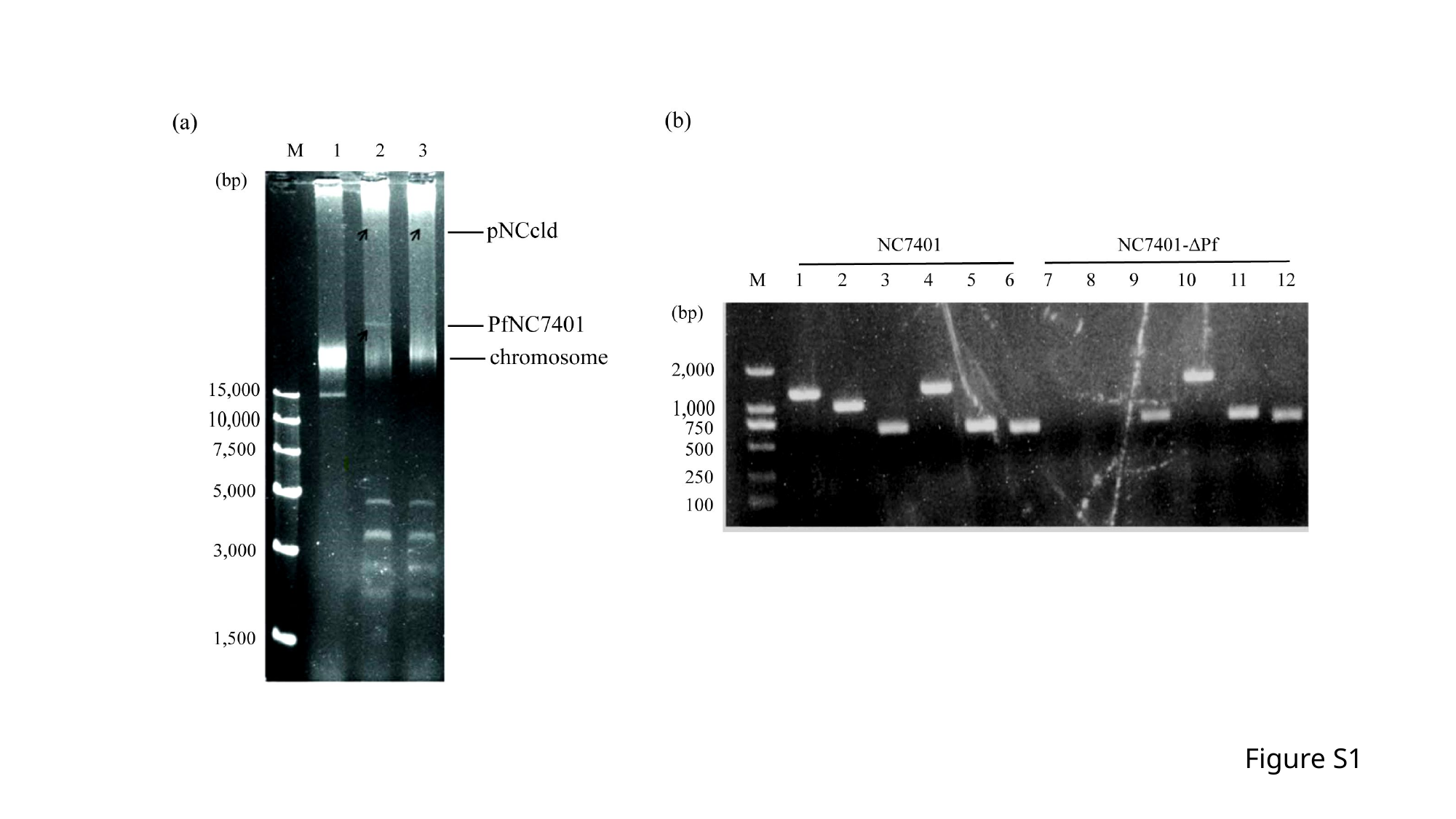

Figure S1

## Slide 2
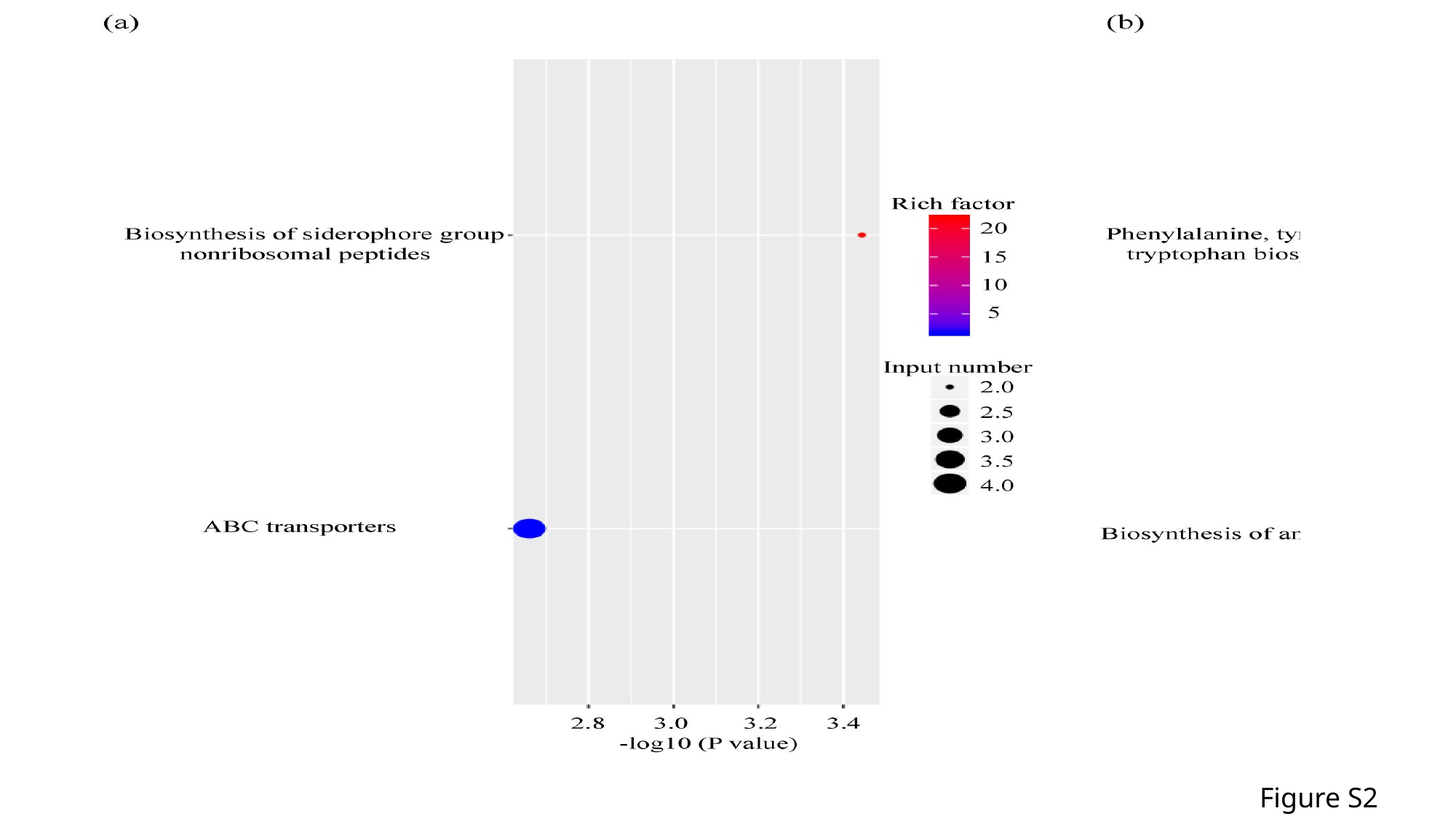

Figure S2

## Slide 3
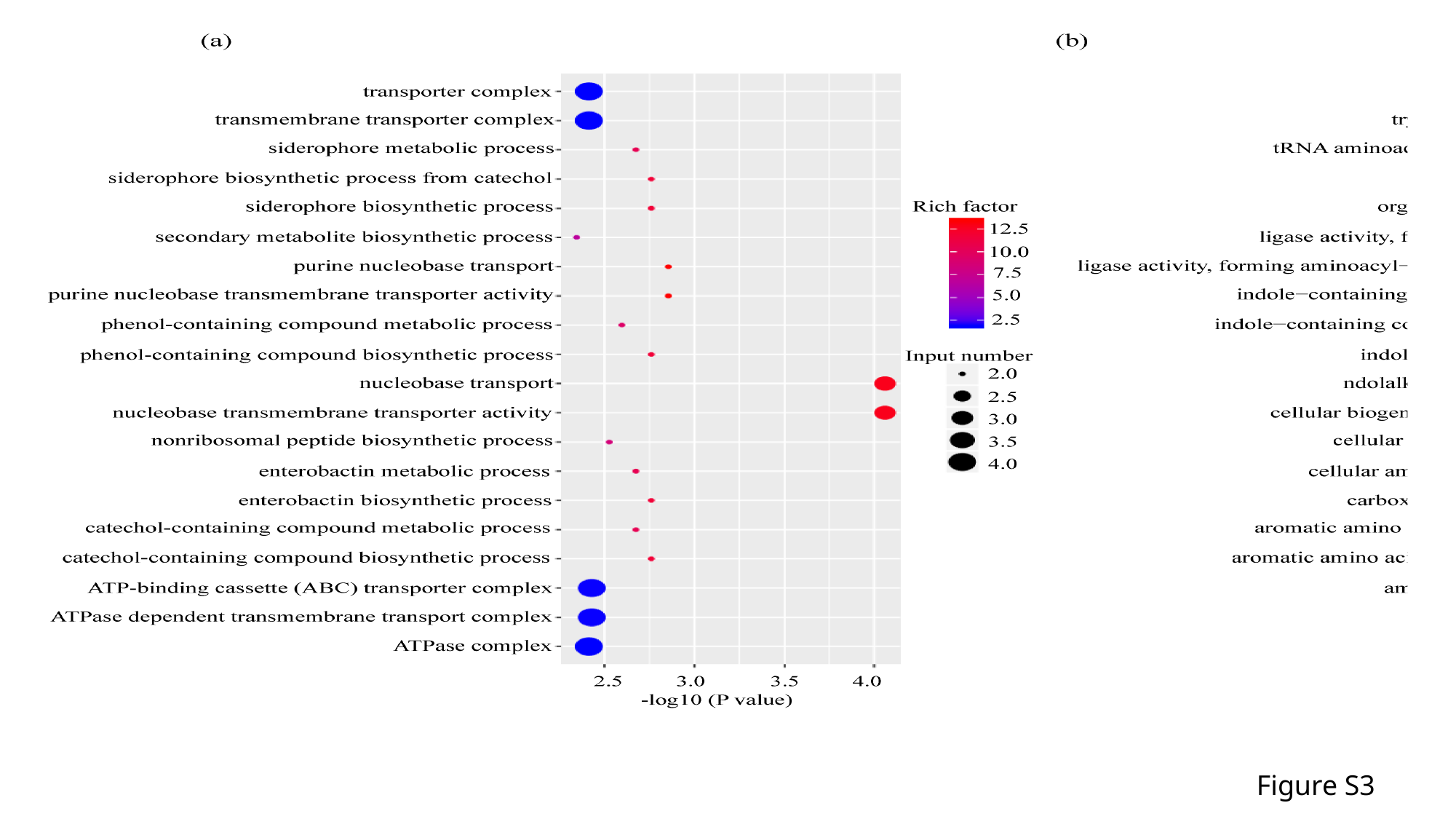

Figure S3

## Slide 4
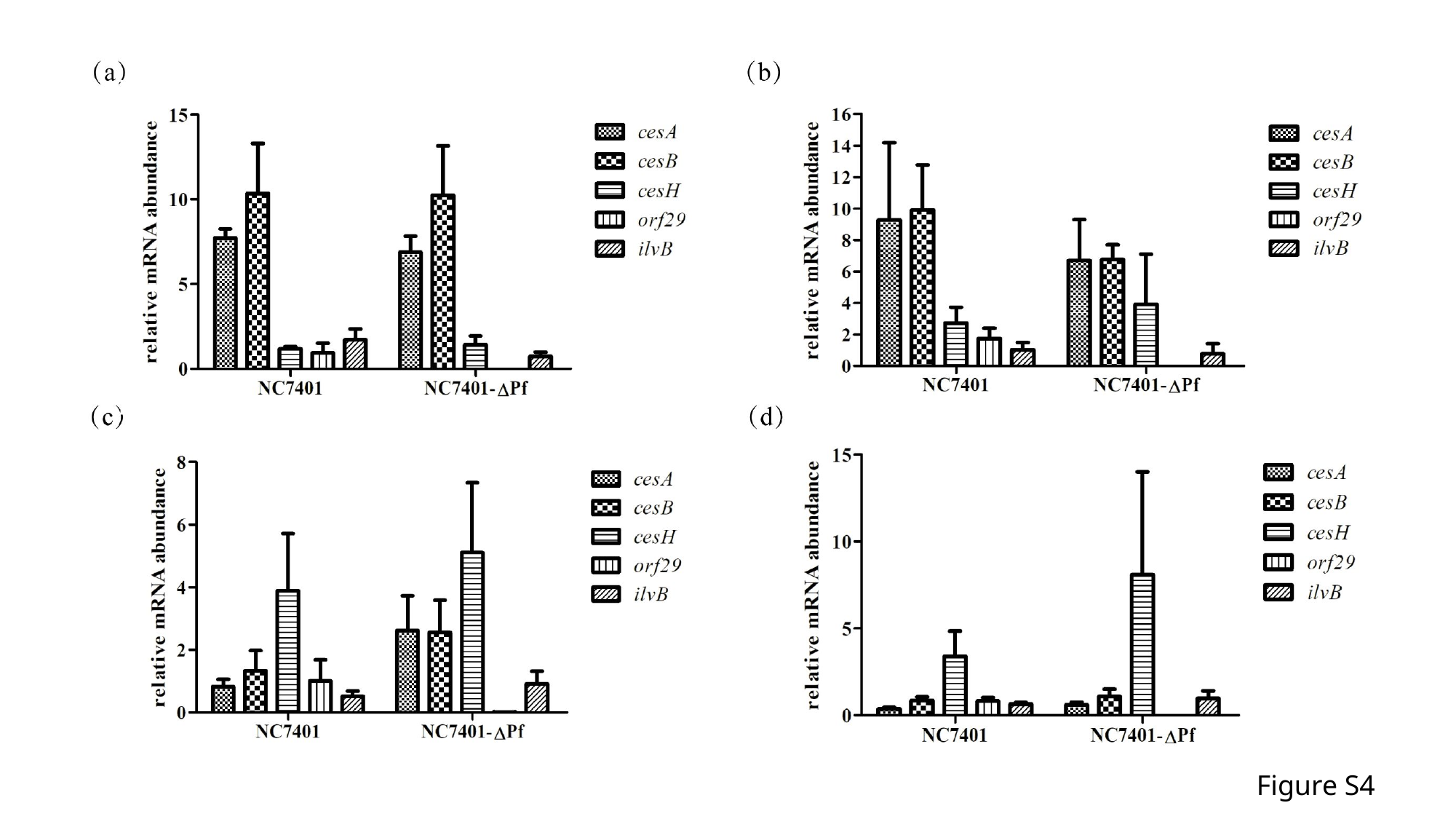

Figure S4

## Slide 5
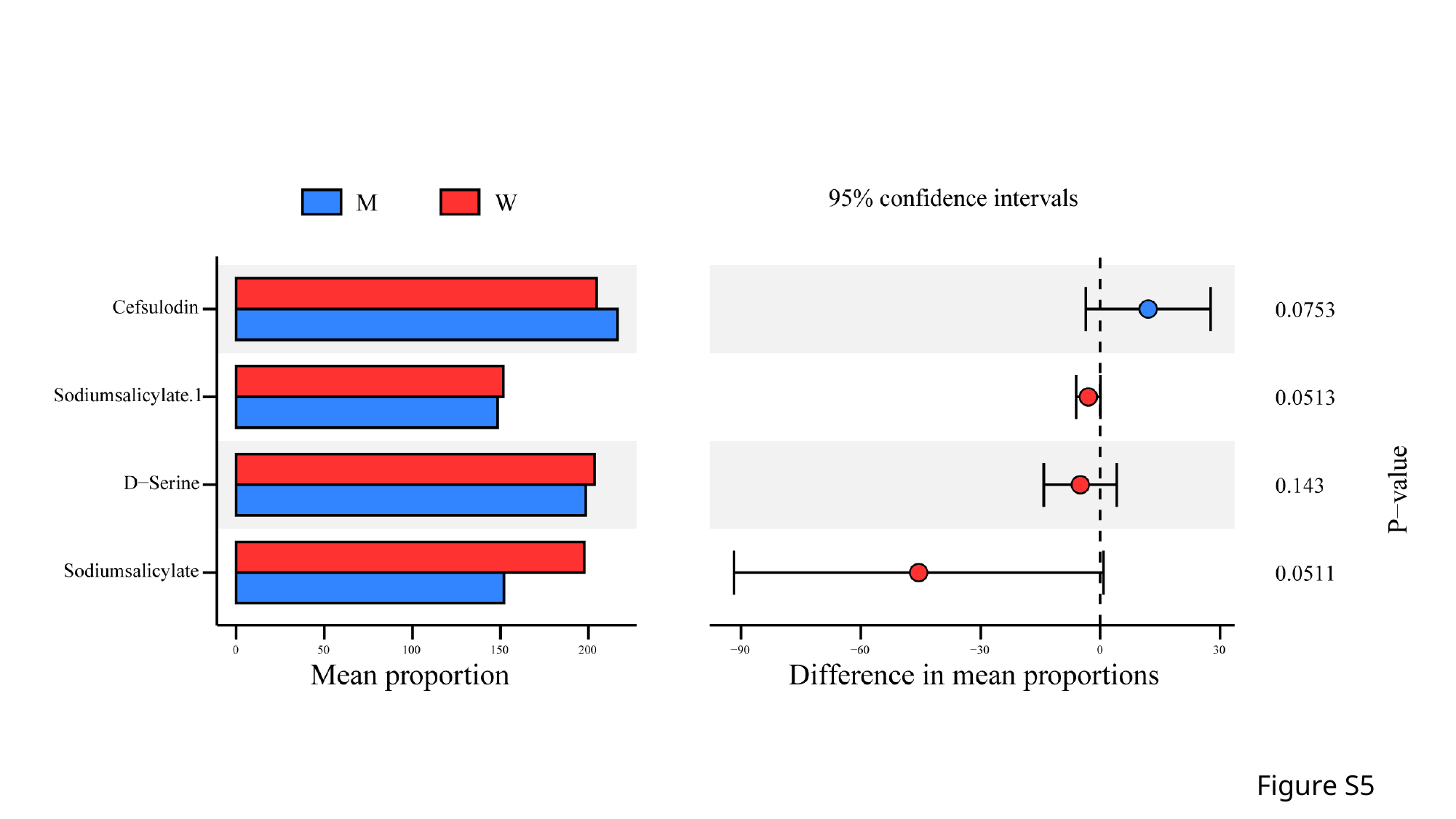

Figure S5

## Slide 6
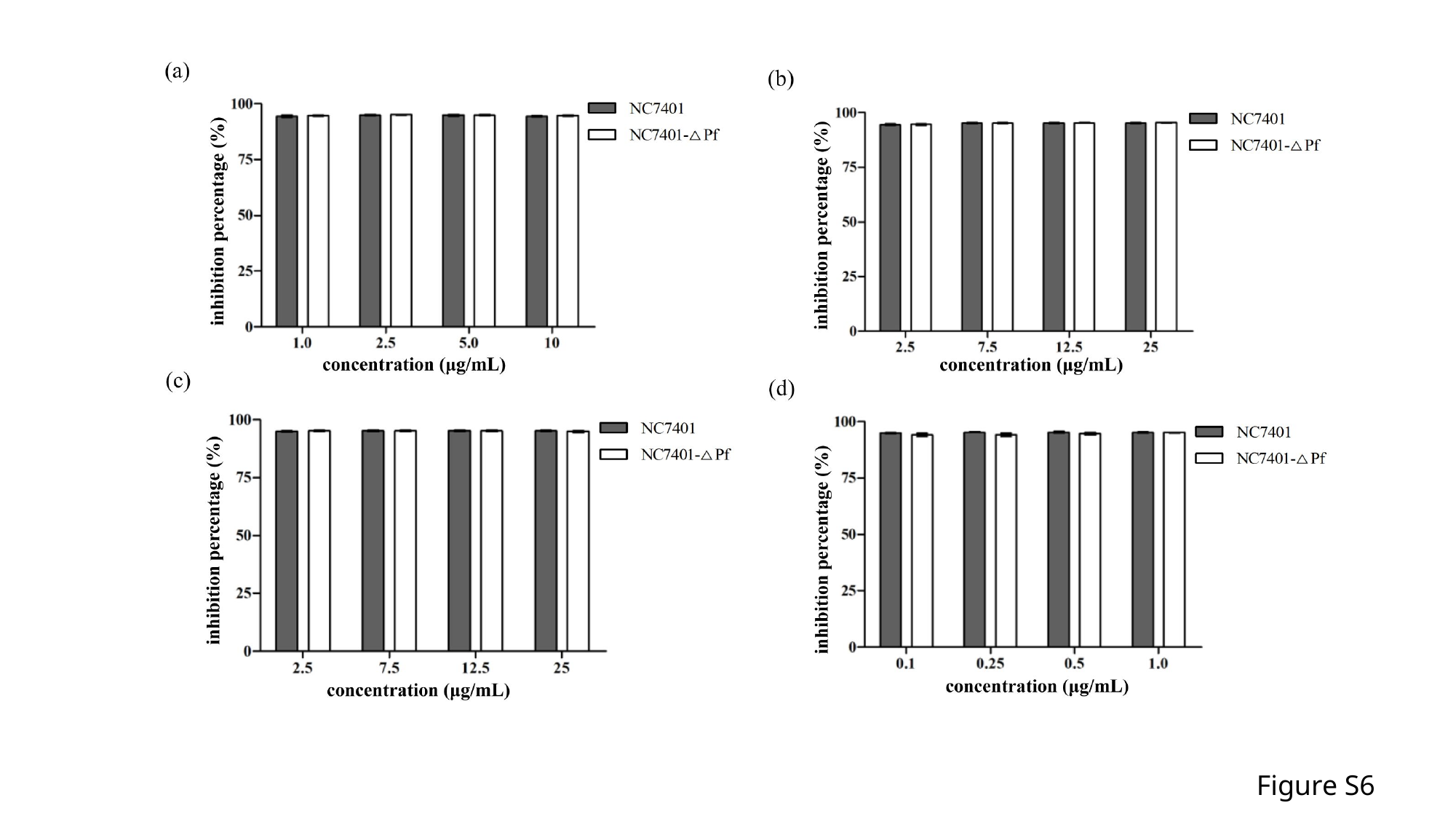

Figure S6

Supplement: Supplementary file 1 [file microorganisms-10-00953-s001.zip › Supplementary figures-edited 20220410 NEW.pptx]
